# Draft Genome Sequence of the Interspecies Hybrid Saccharomyces pastorianus Strain HA2560, Isolated from a Municipal Wastewater Treatment Plant

**DOI:** 10.1128/genomeA.00341-18

**Published:** 2018-04-26

**Authors:** Hakim Tafer, Katja Sterflinger, Ksenija Lopandic

**Affiliations:** a VIBT-Extremophile Center, University of Natural Resources and Life Sciences Vienna, Vienna, Austria

## Abstract

Saccharomyces pastorianus is an industrially relevant yeast frequently isolated from brewing environments. Here, we report the draft genome sequence of the S. pastorianus HA2560 strain isolated from a municipal wastewater treatment plant.

## GENOME ANNOUNCEMENT

Lager beer or bottom-fermenting yeasts, which are taxonomically classified as Saccharomyces pastorianus (synonym Saccharomyces carlsbergensis), arose through the hybridization of Saccharomyces cerevisiae and a cold-tolerant Saccharomyces eubayanus strain (1). These industrially significant yeasts comprise two genetically and metabolically heterogeneous groups, Saaz and Frohberg, that differ in the proportions of their S. cerevisiae and S. eubayanus subgenomes, as well as in the efficiency of their maltotriose utilization and adaptation to cold fermentation temperatures (2–4). The strains of the Saaz group have a reduced S. cerevisiae genomic moiety, are usually unable to utilize maltotriose, and are better adapted to cold than the strains of the Frohberg group. Whole-genome sequences show that the reference strain CBS1513 of the Saaz group is an allotriploid strain with an estimated genome size of 19.36 Mb, whereas the Frohberg strain Weihenstephan WS34/79 is an allotetraploid strain of ∼22.96 Mb (3, 5).

The HA2560 yeast strain, isolated from a municipal wastewater treatment plant in Ulaanbaatar, Mongolia, proved to be related to a lager beer S. pastorianus hybrid (6). However, different molecular markers showed that the genome of the HA2560 strain might be different to some extent from those of the other lager yeasts, indicating that the specific environment from which the strain was isolated might influence its genome shaping. In order to deepen our study, we generated the whole-genome sequence of the HA2560 strain. This sequence can help us determine the genomic constitution of the HA2560 hybrid strain and genetic variations in comparison to the other S. pastorianus yeasts, as well as the influence of different selective pressures on genome evolution. Genome sequencing was carried out using Ion Torrent technology (Ion PGM Hi-Q View kit; Life Technologies, Inc., Carlsbad, CA, USA), according to the manufacturer’s protocols. A total of 1.38 Gb, with a median read length of 260 bp, was generated and assembled with Newbler version 2.9 into a 22.98-Mb genome (∼60.0× coverage) containing 943 contigs (*N*_50_, 60,082 bp). This genome size is similar to the previously estimated genome size of 22.96 Mb of the WS34/70 strain (3), suggesting that the HA2560 strain is a Frohberg S. pastorianus strain. Comparative analysis of the HA2560 genome sequence with the sequence of the WS34/70 (assembly AZAA01) strain showed a coverage of 15.46 Mb (67.3%).

### Accession number(s).

The whole-genome shotgun project of S. pastorianus HA2560 has been deposited at DDBJ/EMBL/GenBank under the accession number PQMD00000000. The version described in this paper is PQMD01000000.
